# EVALUATION OF THE SPATIAL RESOLUTION IN MONTE CARLO-BASED SPECT/CT RECONSTRUCTION OF ^111^IN-OCTREOTIDE IMAGES

**DOI:** 10.1093/rpd/ncab055

**Published:** 2021-04-22

**Authors:** Emma Wikberg, Martijn van Essen, Tobias Rydén, Johanna Svensson, Peter Gjertsson, Peter Bernhardt

**Affiliations:** 1 Department of Medical Radiation Sciences, Sahlgrenska Academy, Gothenburg University, Sahlgrenska University Hospital, 413 45, Gothenburg, Sweden; 2 Department of Clinical Physiology, Sahlgrenska University Hospital, 413 45, Gothenburg, Sweden; 3 Medical Physics and Medical Bioengineering, Sahlgrenska University Hospital, 413 45, Gothenburg, Sweden; 4 Department of Oncology, Sahlgrenska Academy, Gothenburg University, Sahlgrenska University Hospital, 413 45, Gothenburg, Sweden

## Abstract

The purpose was to evaluate the spatial resolution in ^111^In-octreotide single-photom emission computed tomography (SPECT)/computed tomography (CT) imaging following reconstructions with three different ordered subset expectation maximizations (OSEM) reconstruction algorithms; attenuation corrected (AC) OSEM, AC OSEM with resolution recovery (ACRR OSEM) and Monte Carlo-based OSEM reconstruction (MC OSEM). SPECT/CT imaging of a triple line phantom containing ^111^In in air and water was performed. The spatial resolution, represented by the full width at half maximum (FWHM) of a line profile, was determined for each line, for X and Y direction and for all reconstructions. The mean FWHM was 12.2 mm (±standard deviation [SD] 3.7 mm) for AC OSEM, 9.3 mm (±SD 2.5 mm) for ACRR OSEM and 8.2 mm (±SD 2.0 mm) for MC OSEM. MC-based SPECT/CT reconstruction clearly improves the spatial resolution in ^111^In-octreotide imaging and since MC simulations can be performed for all photon energies MC OSEM has the potential to improve SPECT/CT imaging overall.

## INTRODUCTION

In patients with cancer, early detection of metastases is crucial for patient survival, because the presence of metastases indicates a more advanced disease and will directly influence the mode of treatment. Consequently, potential curative treatment options require accurate and early detection of metastases^(1, 2)^.

Nuclear medicine imaging with single-photon emission computed tomography (SPECT) or positron emission tomography (PET) cameras is part of cancer diagnostics. These images, however, suffer from reduced image quality because of the camera design, photon attenuation and scattering. For SPECT images in particular, these effects cause poor resolution, low contrast, depth dependence and high noise levels. Accurate attenuation correction is nowadays standard, but scatter correction is more challenging to perform accurately^(3)^. By simulating photon attenuation and scattering in the body and in the detector with the Monte Carlo (MC) techniques, the limitations mentioned can be accounted for and the image quality can be improved^(4, 5)^.

Neuroendocrine tumours (NETs) have traditionally been visualised with somatostatin receptor scintigraphy, planar as well as SPECT imaging with indium-111 (^111^In) octreotide. The method utilises the overexpression of somatostatin receptors on the tumour cell surface. In recent years, scintigraphy with ^111^In-octreotide has at many sites been replaced by PET imaging with somatostatin analogues labelled with Gallium-68 (^68^Ga), which yields better image quality^(6)^ and higher diagnostic sensitivity^(7)^. A considerable number of studies have concluded the superiority of ^68^Ga PET/CT over ^111^In-octreotide SPECT/CT^(8–10)^. However, the distribution of PET cameras worldwide is not nearly as extensive as that of SPECT cameras^(11, 12)^. Although Buchmann *et al*.^(8)^ concluded that the detection of NET manifestations in the liver and brain is similar for ^68^Ga PET and ^111^In SPECT, consequently there is a need for improvement of the image quality in ^111^In-octreotide SPECT/CT imaging.

**Figure 1:**
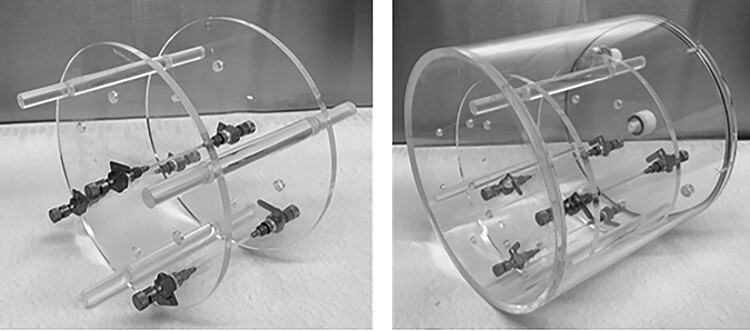
The triple line insert (to the left) and Jaszczak phantom with triple line insert (to the right).

By implementing MC simulations into the ordered subset expectation maximisation (OSEM) reconstruction of SPECT images the image quality can be improved which has been shown in several studies^(4, 5)^. Yet, these simulations are often far too time-consuming to be used in the clinical setting. However, Ryden *et al*.^(4)^ showed that simulations can be parallelized using graphics processing units (GPUs) instead of the central processing unit that resulted in high-quality clinical images within 3 minutes; they presented clearly improved SPECT image quality in phantom measurements with lutetium 177 (^177^Lu). The code presented by Ryden *et al*.^(4)^, the Sahlgrenska Academy reconstruction code (SARec), is used in the present study.

More accurate and timeous diagnosis would result in a more favourable prognosis for the patient as well as lower costs for the healthcare system. The purpose of this study was to quantitively evaluate and compare the spatial resolution for three different reconstruction methods in ^111^In-octreotide SPECT/CT imaging. The reconstruction methods used were conventional attenuation corrected (AC) OSEM, AC OSEM with resolution recovery (ACRR OSEM), where the resolution recovery method used was General Electrics (GE, General Electric Medical Systems, Milwaukee, WI, USA) correction for point spread function (PSF) called Evolution, and MC-based OSEM reconstruction (MC OSEM) with SARec.

## MATERIALS AND METHODS

The reconstruction code used in this study uses MC simulations in the forward projection of an OSEM iterative process^(4)^. The use of GPUs enables acceptable reconstruction times, which makes it applicable in the clinic setting. The code has been improved since publication, and, in this study, it also includes correction for the PSF in the back projection of the iterative process of the OSEM reconstruction.

**Figure 2:**
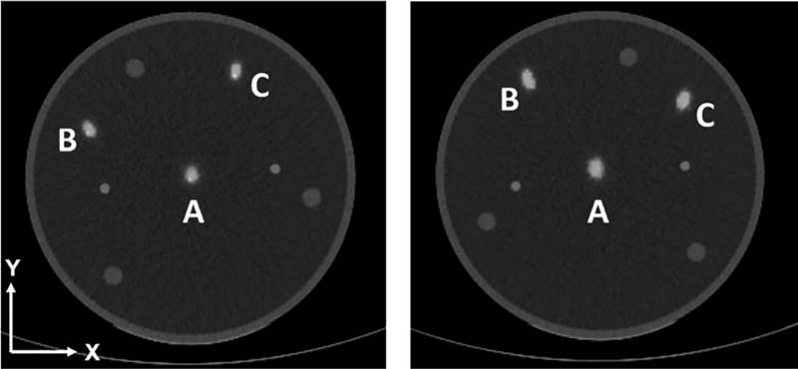
Axial images reconstructed with MC OSEM from camera 1 (left) and camera 2 (right). The three line sources have been labelled according to their position, A, B and C.

A triple line phantom was filled with a solution of ^111^In-octreotide. The inner diameter of the lines was ~ 1 mm and the material was poly (methyl methacrylate)(PMMA). The triple line phantom is an insert to the Jaszczak phantom (Figure 1) and measurements were performed with the triple line phantom itself (in air) and as an insert, after which the Jaszczak phantom was filled with water. SPECT/CT was performed with two cameras, in this study called camera 1 and camera 2, both of model Discovery 670 Pro from GE. The crystal thickness was 15.9 mm, and the collimators used were medium energy parallel-hole collimators. Two 20% energy windows were used, centred around the 171 and 245 keV photon peak, respectively. The matrix size was 256 × 256 with a pixel size of 2.21 mm. The image acquisition was performed with 120 projections and 30 seconds frame duration. Reconstructions were made with three different methods, conventional AC OSEM, ACRR OSEM (Evolution from GE) and MC OSEM. Reconstructions with AC OSEM and ACRR OSEM were performed with software Xeleris 4.0 from GE and clinical settings (at Sahlgrenska University Hospital, Gothenburg, Sweden) with 2 iterations and 10 subsets were used. For MC OSEM, 5 iterations and 10 subsets were used^(4)^. Line profiles were drawn in the X (anteroposterior axis) and Y (left–right axis) direction in the transverse plane (see Figure 2 for representation of X and Y direction) for each line and for both cameras. The spatial resolution was represented by the full width at half maximum (FWHM) of a Gaussian fit of the line profile (measurements were performed with MATLAB 2019b).

To visualise the impact that improved resolution might have in liver tumour diagnosis, a metastasis was simulated into the SPECT raw data of a confirmed healthy patient. The matrix size was 128 × 128 with a pixel size of 4.42 mm and a slice thickness of 4.42 mm. The simulation was performed by randomly placing a sphere of pre-determined size and tumour to normal tissue activity concentration (TNC) ratio inside the liver volume of interest (VOI). The sphere was 1 voxel in radius, and the TNC ratio was set to 8. The raw data of the sphere were simulated using MC simulation of an ^111^In-octreotide SPECT/CT examination (120 projections) and was added to the healthy patient’s raw data. Reconstructions were made from the summarised raw data with AC OSEM and MC OSEM. Unfortunately, we were not able to reconstruct the manipulated raw data with ACRR OSEM due to limitations in the software from GE and to the fact that the method is not publicly available. A Butterworth post filter (order of 10 and critical frequency 0.48 cycles/cm) was used for AC OSEM since this is what is often used in the clinical setting at Sahlgrenska University Hospital. The visibility of the metastasis was compared between the two methods, both visually and by measuring the TNC ratio in the final images. The TNC ratio was determined by placing a VOI over the tumour (total size 4 voxels) and another VOI, representing the normal tissue, surrounding the tumour VOI (of range 1 voxel in each of the three dimensions, total size 15 voxels). The mean counts in the tumour VOI were then divided by the mean counts in the surrounding VOI.

### Statistics

For the quantitative evaluation, the data were analysed using the paired Student’s *t*-test. The statistical tests were performed in IBM SPSS Statistics version 27 (IBM Corporation, Armonk, New York, USA). A *P* value <0.05 was considered to indicate statistical significance.

## RESULTS

The overall spatial resolution (± standard deviation, SD) of the line sources is presented as a mean of both cameras, all three line sources and also as a mean of the X and Y direction. The spatial resolution in water was 12.2 (±3.7) mm for AC OSEM, 9.3 (±2.5) mm for ACRR OSEM and 8.2 (±2.0) mm for MC OSEM. In the reconstructions of the triple line phantom in air, no attenuation correction was made in any of the reconstruction methods; therefore, they are named non-corrected (NC) OSEM, NCRR OSEM and MC NC OSEM, respectively. The spatial resolution for the triple line phantom in air was 13.6 (±3.3) mm for NC OSEM, 9.1 (±1.7) mm for NCRR OSEM and 8.7 (±1.4) mm for MC NC OSEM. The overall results for the measurements in air and water can be seen in Table 1. The spatial resolution in air and water are in agreement quantitively, also when analysed further, and therefore the following presentation of the results will mainly focus on the measurements in water.

**Table 1:** The overall mean spatial resolution for both cameras, all line sources and the X and Y direction for the measurements in water and air, respectively.

Mean resolution ± SD [mm]	In water	In air
AC OSEM/NC OSEM	12.2 ± 3.7	13.6 ± 3.3
ACRR OSEM/NCRR OSEM	9.3 ± 2.5	9.1 ± 1.7
MC OSEM/MC NC OSEM	8.2 ± 2.0	8.7 ± 1.4

**Figure 3:**
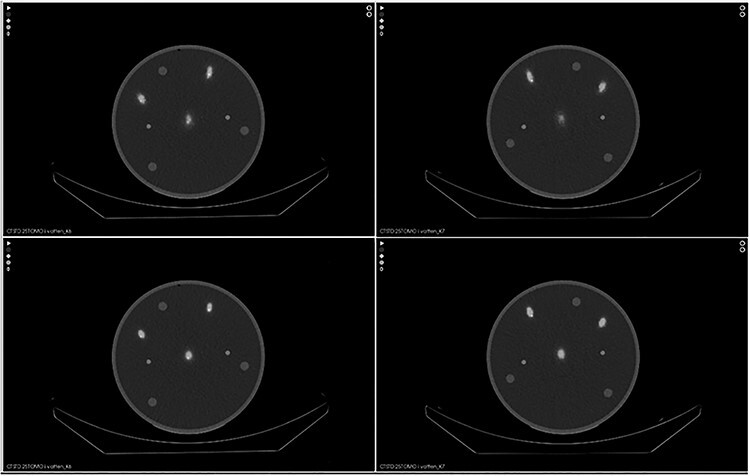
Axial images reconstructed with AC OSEM (upper row) and ACRR OSEM (lower row). To the left are images from camera 1 and to the right from camera 2. The line sources are named according to Figure 2 for each camera respectively.

**Figure 4:**
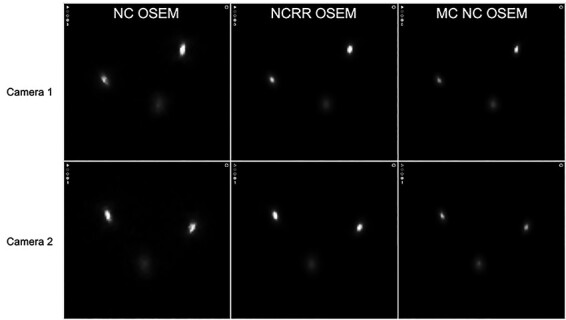
Axial images from reconstructions of measurements in water without attenuation correction with camera 1 (upper row) and camera 2 (lower row). From left to right: NC OSEM, NCRR OSEM and MC NC OSEM.

**Figure 5:**
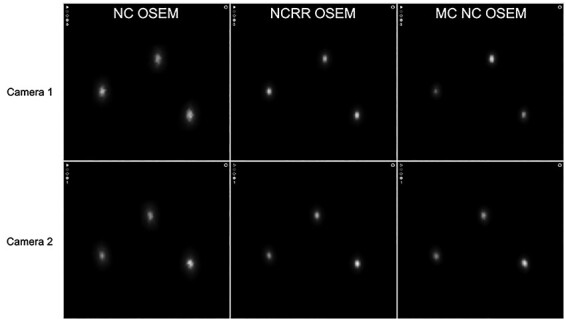
Axial images from reconstructions of measurements in air with camera 1 (upper row) and camera 2 (lower row). From left to right: NC OSEM, NCRR OSEM and MC NC OSEM.

For the measurements in water, there was an 11.2% improvement in spatial resolution with MC OSEM compared with ACRR OSEM and a 32.7% improvement compared with conventional AC OSEM. The difference in the mean spatial resolution between the methods is statistically significant for MC OSEM compared with ACRR OSEM (*P* < .001; 95% confidence interval [CI] 0.6–1.5) and for MC OSEM compared with AC OSEM (*P* < .001; 95% CI 2.8–5.1). There is, however, a clear difference in the X and Y direction for all reconstruction methods and for both cameras (Table 2). This is true also for the measurements in air. It can be seen in Figure 2 for MC OSEM and in Figure 3 for AC OSEM and ACRR OSEM. (Figure 2 also presents the positioning of the line sources and their corresponding labels.) The cylindrical shaped line sources appear elliptical instead of circular in the transverse plane (axial images). The ellipses point towards the centre of rotation (CoR) which is located ~2 cm below line source A. The appearance remained even if the attenuation correction was removed (Figure 4). There is, however, a visual difference in the measurements in air, most prominent for AC OSEM. The elliptical shape is still present, but the direction of the ellipses is more in the anteroposterior direction than towards the centre (Figure 5). The results of the quantitative measurements for camera 1 and 2 are presented in Table 2. The relationship between the reconstruction methods is essentially maintained weather observing the X or Y direction (MC OSEM has a better spatial resolution compared with the other two reconstruction methods). The differences between the X and Y direction are significant for all reconstruction methods (Table 3). The difference between the X and Y direction is higher for AC OSEM than for the other two reconstruction methods, as observed visually in Figure 5.

**Table 2:** The spatial resolution in water of the three line sources (A–C) imaged with camera 1 and 2. Resolutions are presented for the X and Y direction separately and for all reconstruction methods.

Resolution [mm]	Camera 1				Camera 2			
	A	B	C	Mean ± SD	A	B	C	Mean ± SD
X direction								
AC OSEM	11.8	9.3	6.8	9.3 ± 2.5	12.8	7.4	9.1	9.8 ± 2.8
ACRR OSEM	9.9	7.1	5.7	7.6 ± 2.1	10.4	6.2	7.0	7.9 ± 2.3
MC OSEM	8.4	6.4	5.0	6.6 ± 1.7	8.7	5.6	6.5	6.9 ± 1.6
Y direction								
AC OSEM	18.0	12.1	14.2	14.8 ± 3.0	18.6	13.6	13.0	15.0 ± 3.1
ACRR OSEM	13.1	9.0	9.7	10.6 ± 2.2	13.8	9.9	9.3	11.0 ± 2.4
MC OSEM	11.1	8.6	8.9	9.6 ± 1.3	11.4	9.1	9.0	9.8 ± 1.3

**Table 3:** The mean differences in spatial resolution for the X and Y direction of camera 1 and 2 for the three different reconstruction methods.

Difference	Mean ± SD [mm]	Mean [%]	*P*	95% CI
AC OSEM	5.4 ± 1.7	60	0.001	[3.6, 7.1]
ACRR OSEM	3.1 ± 0.9	43	<0.001	[2.2, 4.0]
MC OSEM	2.9 ± 0.6	46	<0.001	[2.3, 3.6]

**Figure 6:**
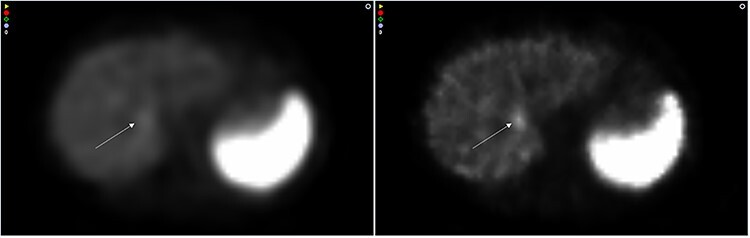
Images of a healthy patient with a simulated metastasis (denoted with an arrow) reconstructed with AC OSEM (left) and MC OSEM (right).

The results from the reconstructions of the simulated metastasis in the healthy patient can be seen in Figure 6. Visually, it is clear that the metastasis can be seen more clearly in the images reconstructed with MC OSEM. Quantitively, the TNC ratio was 1.07 with AC OSEM compared with 1.33 with MC OSEM.

## DISCUSSION

As expected, the spatial resolution is improved with MC-based SPECT/CT reconstruction. This has been demonstrated before by Ryden *et al*.^(4)^. However, this study is the first study to establish this result in ^111^In-octreotate SPECT/CT imaging, and by using line sources that was not used by Ryden *et al*. It can be concluded that it has an additional value to not only compensate for the point spread effect but to fully simulate the photons’ path through the CT and the collimator to the crystal.

**Figure 7:**
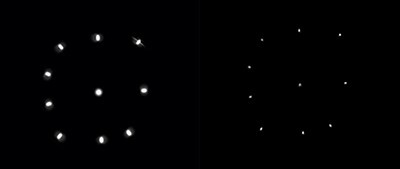
Axial images of simulated line sources in air reconstructed with NC OSEM (left) and MC OSEM (right).

The differences in X and Y directions can be seen visually in the images as the cylindrical shaped line sources appear elliptical instead of circular in the transverse plane. The ellipses seem to point towards the CoR, which is located ~2 cm below line source A. This phenomenon has been observed before in imaging with the National Electrical Manufacturers Association (NEMA) image quality phantom as well as the Jaszczak phantom with different radionuclides but this has not been addressed or explained before^(13–16)^. The appearance is the same if the attenuation correction is removed but in the images from the measurements in air, the ellipses have more of an anteroposterior direction. The appearance is the same for both cameras but also for all reconstruction methods. Simulations, that were performed in order to further analyse the problem, showed that the PSF corrections in ACRR OSEM and MC OSEM does improve the appearance but both reconstruction methods are unable to fully image the line sources correctly (see comparison between NC OSEM and MC OSEM in Figure 7). This conclusion is supported quantitatively by the differences in mean spatial resolution between the X and Y direction where AC OSEM has a larger difference than the other reconstruction methods (Table 3). The improvement can also be seen visually in Figures 2–5. The number of iterations did not have an impact on the appearance (data not shown). In this study, the images of the line sources are all unfiltered. Very often, a smoothing post filter is applied after reconstruction to limit the noise, and these filters seem to eliminate the appearance that is observed in this study. Regardless, this phenomenon could possibly contribute to inaccurate determination of the activity distribution in SPECT/CT imaging.

Regarding the simulated metastasis in the healthy patient, there is a clear improvement in spatial resolution, which has also been shown quantitatively in this study, and also a clear improvement of the contrast, which is quantitatively established with the higher TNC ratio.

Clinical comparisons between SPECT and PET performed by Buchmann *et al*.^(8)^ revealed different results for different regions. The detection of NETs in the liver was equal between the two examinations. The authors report the use of iterative reconstruction using the OSEM algorithm for the ^111^In SPECT imaging and do not specify further, thus indicating that no corrections for PSF, scatter or even attenuation were made. Among the references, in this study, comparing ^111^In-SPECT and ^68^Ga-PET, only one^(6)^ has reported the use of attenuation and PSF correction, remaining authors have not reported any corrections at all, indicating that many comparisons may not have been completely fair. Furthermore, the field of SPECT is developing with new camera designs^(17, 18)^ as well as improved reconstruction techniques. Based on the results in this study, a comparison between ^68^Ga PET/CT and MC-based reconstruction of ^111^In-octreotide SPECT/CT would be of interest to evaluate, both regarding image quality and diagnostic sensitivity. A limitation of this paper is that it does not address the aspect of the detection of low contrast structure or the impact on quantification. This will be included in future studies. Furthermore, decreasing the noise level in ^111^In SPECT/CT imaging by adding synthetic intermediate projections, which was successfully accomplished for ^177^Lu SPECT images^(19)^, would also be relevant to explore further for ^111^In SPECT images.

## CONCLUSION

MC-based SPECT/CT reconstruction clearly improves the image quality significantly in ^111^In-octreotide imaging. With reconstruction times of just a few minutes, it would now be possible to implement in the clinic and thereby improve the image quality for both ^177^Lu and ^111^In SPECT/CT imaging. A simulated metastasis in a healthy patient has been demonstrated to be better visualised with MC-based SPECT/CT reconstruction compared with AC OSEM reconstruction.
